# Identification of Antifreeze Proteins and Their Functional Residues by Support Vector Machine and Genetic Algorithms based on *n*-Peptide Compositions

**DOI:** 10.1371/journal.pone.0020445

**Published:** 2011-05-31

**Authors:** Chin-Sheng Yu, Chih-Hao Lu

**Affiliations:** 1 Department of Information Engineering and Computer Science, Feng Chia University, Taichung, Taiwan; 2 Master's Program in Biomedical Informatics and Biomedical Engineering, Feng Chia University, Taichung, Taiwan; 3 Graduate Institute of Molecular Systems Biomedicine, China Medical University, Taichung, Taiwan; National Institute for Medical Research, Medical Research Council, London, United Kingdom

## Abstract

For the first time, multiple sets of *n*-peptide compositions from antifreeze protein (AFP) sequences of various cold-adapted fish and insects were analyzed using support vector machine and genetic algorithms. The identification of AFPs is difficult because they exist as evolutionarily divergent types, and because their sequences and structures are present in limited numbers in currently available databases. Our results reveal that it is feasible to identify the shared sequential features among the various structural types of AFPs. Moreover, we were able to identify residues involved in ice binding without requiring knowledge of the three-dimensional structures of these AFPs. This approach should be useful for genomic and proteomic studies involving cold-adapted organisms.

## Introduction

Antifreeze proteins (AFPs) prevent macroscopic ice build-up in cold-adapted organisms by binding to ice and thereby forestalling additional crystallization [1]. By doing so, AFPs allow organisms to survive below 0°C. It is of great interest to harness this singular property—non-antifreeze proteins cannot bind ice—for applications related to the agriculture and food industries [2], [3], [4], [5] and to the rational design of new AFPs. In this regard, it is first necessary to understand how AFPs interact with ice. Accurately identifying AFPs from evolutionarily divergent organisms is difficult because their sequences and structures differ radically [6], [7]. To complicate matters further, for closely related species, the sequences of their AFPs (and consequently the structures,) may also differ substantially if they have been geographically isolated [8]. Additionally, searching for homologous sequences within databases has not been fruitful given the disparity among AFP sequences. Directly studying AFP-ice interactions is also difficult, and a definitive picture of such interactions is not currently available [7]. Because many AFPs do not have structural or sequential features in common, it is therefore challenging to correlate the relationships between sequences, structures, and function.

A large number of biochemical and structural studies [9], [10], [11] have been performed in an attempt to understand how AFPs interact with ice at the molecular level, including site-directed mutagenesis experiments [12], [13], [14] and computational approaches [15]. An ice-binding model that incorporates surface complementarity is generally accepted [16]. Recently, Doxey and colleagues [9] successfully identified AFPs, for which three-dimensional (3D) crystallographic structures were available, on the basis of their highly ordered and planar ice-binding surfaces. Their algorithm, however, could not identify AFPs where only NMR solution structures were available, because the coordinates for atoms at and near the surface were not well defined. [9], [17]. Because the algorithm required a 3D crystallographic structure it was somewhat limited, as there are not always available for any given AFP.

It is clear then, that AFPs cannot be easily distinguished from other types of proteins. Additional information is needed to understand how AFPs and ice interact on a fundamental physicochemical level before such interactions can be applied to cold-adapted mechanisms. Although the types of amino acids present are closely linked to the ice-binding properties of AFPs [10], [13], current models usually rely on only 3D structures. Additionally, to make use of the knowledge that has accumulated over the decades, e.g., identification of the “hydrophobic surface” effect [7], [11], the spatial regularity of an AFP solvent accessible surface, the presence of nonpolar residues, and other properties directly related to the binding properties of AFPs, an algorithm that can take these properties into consideration is necessary. Here, we developed an integrated approach to rapidly identify AFPs from their amino acid sequences. When given a query sequence, our statistically based, support vector machine (SVM) algorithm has been used to identify certain inherent protein traits e.g., protein disulfide connectivities [18], subcellular localizations [19], [20], and protein folds [21], and it does not require a computational mechanical model or structure comparison. During the training and testing of this algorithm for different classifiers associated with AFPs, multiple feature schemes based on *n*-peptide compositions extracted from the sequences were used. Then, a genetic algorithm (GA) was used iteratively for key-feature selection and to improve the identification accuracy. This integrated approach enabled the recognition of AFPs on the basis of preferred short peptide sequences, rather than on structural comparisons. The identified AFP sequence features have not been reported previously, yet they correlate well with the properties of the ice-binding interfaces. This approach is suitable for the further identification of the ice-binding surfaces of AFPs.

## Methods

### The Validation Dataset that Contained AFPs and non-AFPs with Known 3D Structures

To assess our approach without bias, we tested it using a sequence validation dataset that did not contain homologous proteins. To examine the effects of key residues on function, we included only AFPs that had known 3D structures. This set contained 3762 nonredundant non-AFPs and 44 AFPs, collected from the PISCES server [22] and the Protein Data Bank (PDB) [23], respectively. To include as many representative structures as possible, the non-AFPs had <25% pairwise sequence identity (SI), R-factors of 0.25 and a crystallographic resolution of at least 2 Å. The AFP sequences were separated into seven subsets on the basis of sequence identity by ClustalW2 [24]. Table 1 lists the PDB IDs of the AFPs in each subset. For a given subset, the associated AFPs had sequences that were not homologous to any of the AFPs in the other subsets. The non-AFPs were randomly divided among the seven subsets to cross-validate the performance of our approach and then were merged as a single trained model for use with other (independent) datasets (see below). Under such a stringent condition, any AFPs recognized subsequently could not have arisen from the self-trained sequences.

**Table 1:** The seven AFP subsets used for cross-validation testing.

Subset	Type	PDB ID
1	insect AFP	1c3y
2	Type III fish AFP	1c89; 3nla; 1ucs; 1ops; 1kde; 1ame; 1msi; 1b7i; 1b7j; 1b7k; 1ekl; 1gzi; 1hg7; 1jab; 1msj; 2ame; 2jia; 2msi; 2msj; 2spg; 3ame; 3msi; 4ame; 4msi; 5msi; 6ame; 6msi; 7ame; 7msi; 8ame; 8msi; 9ame; 9msi;
3	β-helical insect AFP	1ezg
4	Type I fish AFP	1wfa; 1j5b; 1y03
5	β-helical insect AFP	1eww; 1l0s; 1m8n
6	insect AFP	2pne
7	Type II fish AFP	2py2; 2afp

### Independent Datasets

To test our algorithm after training it with the sequence validation dataset, we constructed three independent datasets; none of the AFPs included in the seven subsets of the validation dataset were included in these three datasets. The first dataset included two AFP structures that had been experimentally verified [9], the second dataset contained 369 nonredundant AFP sequences from the UniProKB database [25], [26], representing an evolutionarily divergent group of organisms, and the third dataset contained two “antifreeze-like” (AFL) proteins—although incapable of binding ice, these have both sequence and structure similarity to the fish type III AFP [27].

The second test dataset was constructed by searching for the phrase “antifreeze” in the UniProtKB database; any redundancies, i.e., duplicate sequence or partial sequences, were removed. Further filtering removed those sequences with “predicted” and “putative” in the protein name field. Proteins were then manually checked against the literature to identify those with an appropriate habitat of the host organism, e.g., Arctic Ocean, desert, or mountain climate. Any proteins belong to “antifreeze-like proteins” were also excluded to avoid confusion. Table 2 lists the number of AFPs from each type of organism included in the second dataset.

**Table 2:** Distribution of the 369 AFP sequences between the types of organism in the independent dataset.

Organism	Number of sequences
Algae	17
Bacteria	101
Fish	123
Insects	105
Plants	23

### Feature based coding schemes

*n*-peptide composition and feature-based coding schemes (where *n* = 1, 2, 3, etc., encodes the amino acid, dipeptide, and tripeptide composition, respectively) have been used to predict protein properties [19], [20], [21], [28]. Here we used them to characterize the important ice-binding features of AFPs. In this method, feature schemes are denoted by the following set of symbols: *A_n_*, the original amino acids; *H_n_*, hydrophobicity [29]; *V_n_*, the normalized van der Waals volume [29]; *Z_n_*, polarizability [29]; *P_n_*, polarity [29]; and *F_n_*, *S_n_*, and *E_n_*, groups of residues classified according to four, seven, and eight physical/chemical properties, respectively [19]. To characterize key functional residues more robustly, partitioned subsequences, *g*-gap dipeptides, and local amino acid composition strategies were also included. [19] The partitioned amino acid composition 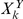 is a concatenation of all amino acid sequences of composition *Y* and length *k*. The symbol *D_g_* identifies the frequency of a sequence in the form *a*(*x*)*_g_b*, where *a* and *b* denote specific amino acids and (*x*)*_g_* denotes the intervening residues of any type between the pair (*g*-gap), where *g* is the number of intervening residue. The symbol *W_l_* indicates the amino acid composition for peptides characterized by a set of sliding windows of length *l* centered on a given type of amino acid. It provides information about the sequential neighbors for of a given type of amino acid.

### Assembly Machine-learning Algorithms

All SVM calculations were performed using LIBSVM [30], a general library for support vector classification and regression, and the radial basis function kernel. In addition to the SVM algorithm [31], we implemented a GA to efficiently optimize the selection of feature attributes as detailed [18]; the combined use these algorithm is denoted as SVMGA. For the SVMGA, the feature attributes of each feature scheme, the penalty parameter C, and the kernel parameter *γ* of the radial basis function (used for SVM identification by the GA approach) were determined in advance. The GA procedure rapidly filtered out feature attributes that were not useful for SVM identification on the basis of each feature scheme.

### The Voting System

The coding scheme symbols given above denote the SVM classifiers that were derived from the various properties of the sequence descriptors. For simplicity, the participants in the SVM-identification system [19], [20] were incorporated as:
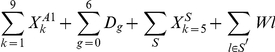
with *S* = {*H_3_*,*V_3_*,*Z_3_*,*P_3_*,*F_3_*,*S_2_*,*E_2_*} and *S*' = {*7*, . . .,*15*}. The system counts the jury votes from each classifier to determine if a protein is an AFP.

### Performance Assessment

As in previous work [19], [20], [21], we calculated the prediction accuracy, *Q_i_*_,_ which is defined as *Q_i_* = *c_i_*/*n_i_* × 100, to assess the performance of identification, where *c_i_* is the number of correctly identified AFPs in the class *i ∈* (AFP, non-AFP), and *n_i_* is the number of sequences. The overall identification accuracy is given by
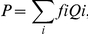
where *f_i_* = *n_i_*/*N*, and *N* is the total number of sequences. Although *Q_i_* provides a convenient assessment for identification performance, the Matthews Correlation Coefficient (*MCC*) [32] is a more informative measure of the performance and is given by:
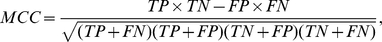
where *TP*, *TN*, *FP*, and *FN* are the number of true positives, true negatives, false positives, and false negatives, respectively. A value for *MCC* of 1, 0, or –1 represents a perfect correlation, a random correlation, or an inverse correlation, respectively. Consideration of the *MCC* allowed us to modify our approach to return as few false positives as possible, thereby maximizing the credibility of the method. To be a credible method, our approach needed to return as few false positives as possible.

### AFP Sequence Homology Search

To verify our ability to identify AFPs from their protein sequences, we tested the homology relationships among the AFP sequences. A query sequence from the second independent data set was aligned with the library sequences of the 44 AFPs of the validation set. Only these 44 AFPs were used because their 3D structures have been solved, and they had been experimentally shown to bind ice. We performed an all-against-all sequence alignment using the global alignment program ALIGN [33]. Only the top-ranked sequence of the 44 AFP sequences was then used to assess the effect of homology on AFP identification, i.e., the SI value for the query sequence and the top-ranked sequence determined the usefulness of the homology search approach.

## Results

### Identification of AFPs in a Cross-validation Dataset

For the cross-validation test, the 3762 non-AFPs were randomly and equally divided amongst the seven subsets of known AFPs, determined on the basis of sequence identity (Table 1). The seven subsets of AFPs can be thought of as seven distant branches of an evolutionary tree. As an experiment, the sequences of six of the subsets were used to train the SVM algorithm with a given feature scheme, and then the output model of the trained algorithm was used to test the sequences in the subset that was not used for training. This training-and-testing cross-validation procedure was repeated seven times for a given feature scheme, each time omitting a different sequence subset during training. All results reported the performance on the total number of datasets. The SVM classifiers were optimized so that the algorithm could assign a protein sequence as either an AFP or non-AFP sequence.

Table 3 contains a summary of the identification accuracies and the *MCC* values for the different combinations of feature schemes; only the best result for a given feature scheme is reported. The optimized overall identification accuracy was around 11% for the SVM algorithm. Incorporation of the GA algorithm dramatically improved the identification accuracy. Using the iterative procedures mentioned above, the GA identified the largest number of *TP*s and the smallest number of *FP*s (<30 *FP*s remained) as it discarded feature attributes that were not useful for the SVM classification. The assembled SVMGA approach correctly identified all of the AFPs in the cross-validation set. Using just the smallest possible number of selected features, the SVM classifier identified a large number of completely structurally dissimilar AFPs than did Doxey et al. [9] who used the structural characteristics of the AFPs.

**Table 3:** Performances of SVM and SVMGA in the seven-fold cross-validation tests.

		SVM	SVMGA	
No. entries	Subset	13 Feature schemes^a^	13 Feature schemes^a^	Doxey et al.[9] b
1	1	0	1	-
33	2	0	33	3
1	3	1	1	1
3	4	0	3	3
3	5	2	3	2
1	6	1	1	-
2	7	1	2	0
AFP accuracy		11.4%	100.0%	90.0%
AFP precision		25.0%	62.9%	42.9%
Overall accuracy		98.6%	99.3%	99.6%
*MCC*		0.162	0.790	0.620
*TP*		5	44	9
*TN*		3747	3736	3184
*FP*		15	26	12
*FN*		39	0	1

a The 13 feature schemes were: 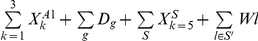 where *k* = {*1*,*5*,*6*,*7*}, *g* = {*0*,*1*,*3*,*6*}, *S* = {*H*_3_,*P*_3_,*S*_2_,}, and *S*' = {7,11}.

b Doxey and colleagues [9] used structure as the property to correctly identify 10 AFPs in their dataset. Only 2atp, based on an NMR structure, was not identified correctly.

### Identification of AFPs in the Independent Datasets

Although the algorithm performed perfectly in determining the number of divergent sequences during the cross-validating process, we sought to apply the algorithm to more realistic datasets. Two AFPs, isolated from the freeze-tolerant winter rye *Secale cereale*, were identified from the first independent dataset: non-specific lipid-transporter protein 1 (LTP1) and LTP2 (UniProtKB codes DQ641934 and DQ641935, respectively). Despite LTP1 and LTP2 having 70% sequence identity, the algorithm accurately distinguished them and identified them as possessing or not possessing antifreeze activity, respectively. In addition to accurately identifying the proteins of the first independent dataset as AFPs, the algorithm also recognized that the human and bacterial AFL proteins (PDB IDs 1wvo and 1xuz, respectively) [27] were not AFPs. The human AFL and the bacterial AFL are both very similar in sequence and structure to that of the fish type III AFP (PDB code 1msi).

**Figure 1:**
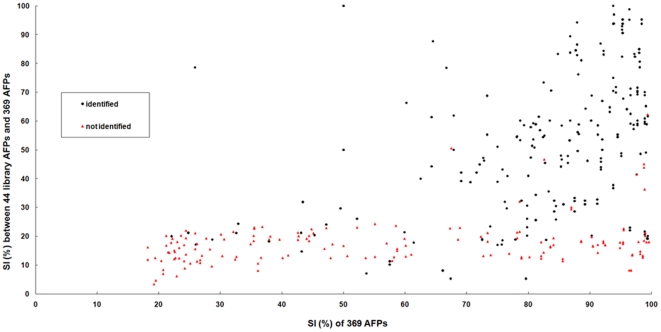
Sequence identity distribution for pairs of AFPs. The *x-*axis values are the best pairwise-matched SI values for each AFP sequence against the other 368 sequences. The *y*-axis values are the best pairwise-matched SI values for each of the 369 AFP sequences of the second independent dataset against the 44 sequences of the validation set. Whether an AFP is identified (black symbol) or not identified (red symbol) in the independent data is indicated.

The AFPs of the second independent dataset represent a divergent group of organisms and were collected from the UniProKB database [25], [26], about 57% of these proteins were identified as AFPs by the SVMGA. The SI pair distribution, which characterizes the relative number of sequence pairs in the close percentage sequence identity interval, was used to examine the effect of sequence homology on AFP identification. The 369 AFP sequences were each used as a query sequence to profile the SI pair-distribution. Each query sequence was aligned with the 44 AFPs of the validation set and also with the other 368 sequences of the second independent data set. The largest SI value for each query that was aligned with the 44 AFPs was plotted along the *y* axis, and the largest SI value for corresponding sequence aligned with the other 368 sequences of the second dataset was plotted along the *x* axis (Figure 1). The SI values associated with AFPs in the independent dataset that were not identified by the SVMGA are shown in red; most of these values are <20%, below the so-called midnight-zone threshold for detection of structural/functional relationship [34]. Because the dataset that contained the 369 AFPs was biased–it only contained AFPs from well-characterized cold-adapted organisms–many of the data points are located at the far end of the *x* axis.

### Coding Schemes

**Figure 2:**
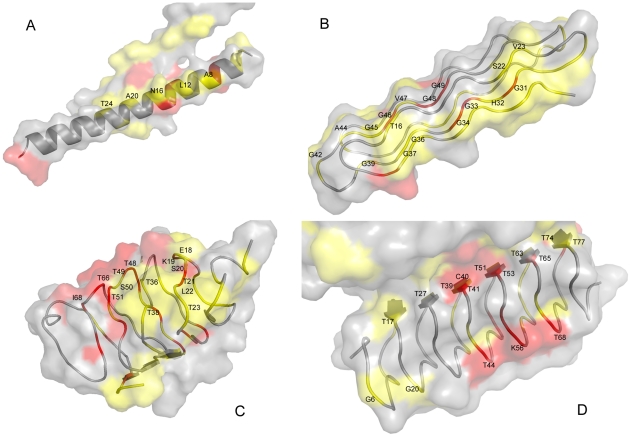
Examples of key residues mapped onto the surfaces of the seven representative AFPs used in the cross-validation tests. The structures were drawn using PyMOL [37]. Identification key residues are denoted in red (more votes) and yellow (fewer votes) for the following PDB structures: (A) the winter flounder α-helical AFP (PDB ID 1wfa) [35]; (B) the snow flea AFP (PDB ID 2pne) [38]; (C) the β-helical spruce budworm AFP (PDB ID 1eww) [13]; (D) the β-helical beetle *Tenebrio molitor* AFP (PDB ID 1ezg) [36].

For the different coding-scheme SVM classifiers used in this study, we were able to reduce the number of feature attributes required by at least 50% after implementing the GA. Consequently, each remaining classifier was well suited to being able to identify the corresponding type of AFP (Table 4). To understand why the features were selected as classifiers, we assigned a number (vote) when the pattern of residues in a sequence matched a GA-selected feature attribute of a coding scheme. The sequence position was marked as an SVMGA key residue if it had received a majority of the jury votes from the 13 coding schemes that we used. For instance, the dipeptide LT was selected in the *D_0_* scheme, and the interval dipeptide T(X_2_)T was selected in the *D_1_* scheme–thus for the short peptide NTALT, the L at the forth position and the first T each received one vote, and the second T received two votes (Table 5). The representative AFPs are presented in Figure 2, and their SVMGA key residues are marked. Residues with >6 votes, with 4 or 5 votes, and with <3 votes are colored red, yellow, and gray, respectively. The average number of SVMGA key residues in AFP sequences and in non-AFP sequences was confirmed as being significantly different. Approximately 70% of the SVMGA-selected key residues are solvent exposed (data not shown), which is as expected because these residues are more likely to interact with ice.

**Table 4:** The feature schemes that enabled the recognition of the AFP in a subset when single SVM classifier was used.

	Feature Scheme									
Subset	*C*	*W_l_*	*D_0_*	*D_1_*	*D_3_*	*S_2_X_5_*	*H_3_X_5_*	*P_3_X_5_*	*V_3_X_5_*	*Z_3_X_5_*
1				•						
2	•	•	•	•	•	•	•	•	•	•
3	•	•	•	•	•					•
4		•	•	•	•	•	•	•		•
5	•	•	•	•	•	•	•	•		•
6		•			•	•	•	•	•	
7			•	•		•				

The filled circles correlate the feature schemes with the AFPs that they identified. The AFPs are denoted according to their subsets.

**Table 5:** An example of votes acquired by residues in a sequence from 1msi.

Sequence		…..	Q^9^	L^10^	I^11^	P^12^	I^13^	N^14^	T^15^	A^16^	L^17^	T^18^	…..
Coding	*C*		*						*			*	
	*X_5_*												
	*X_6_*												
	*X_7_*												
	*D_0_*										*	*	
	*D_1_*		*				*	*	*	*			
	*D_3_*												
	*D_6_*			*				*	**				
	*O_3_X_5_*										*		
	*P_3_X_5_*							*	**	**		*	
	*S_2_X_5_*		*	*					*	*	*	*	
	*W_7_*			*			*	*	*	*			
	*W_11_*					*							
Votes		…..	3	3	0	1	2	4	8	5	3	4	…..

## Discussion

Previous studies have postulated the structural character of the interactions between ice and AFP molecules [7], [14]. Knowing how ice and AFP molecules interact allows for the identification of AFPs from their structures, as demonstrated by Doxey et al. [9] (Table 3). Their method, however, requires 3D protein structures, and until now there has not been a more general method to predict candidate AFPs.

**Figure 3:**
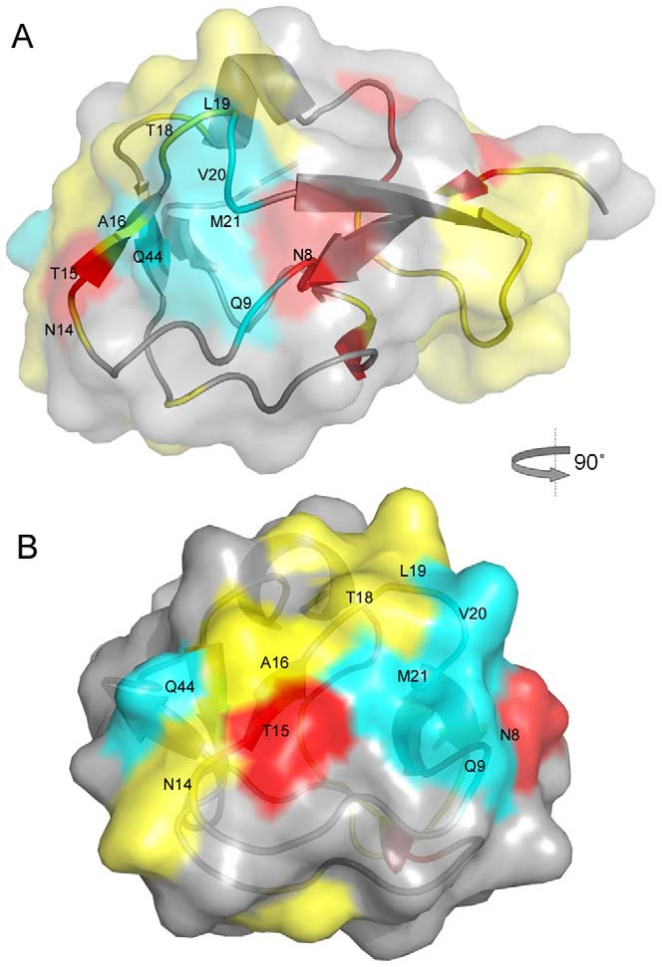
The surface of the eelpout type III AFP (PDB ID 1msi). (A) Key residues selected by the SVMGA are labeled in black words. Residues Q9, V20, M21 and Q44, which were identified as key residues in a mutagenesis study but not by the SVMGA, are shown in cyan. (B) A view of the ice–binding interface; all residues that are part of the interface as reported are labeled. The residues identified by SVMGA are shown in red and yellow. Residues known to be important in ice binding, but not identified by the SVMGA are shown in cyan. Residues not identified by the SVMGA are shown in gray. Residues not determined by SVMGA are shown in gray.

To identify AFPs here, we used an integrated machine-learning method, SVMGA, that uses multiple *n*-peptide composition features. Our results show that sequentially divergent AFPs can be identified according to their shared sequence characteristics, because any test sequence or its homologs are not used in the training set. A set of *n*-peptide, composition-based, SVM predictors were combined to accurately recognize AFPs, and more importantly, to identify the key functional residues neighboring the ice-binding surfaces. Jia and Davies [7] have characterized defining residue repeats in AFP sequences, e.g., alanine-rich α-helix of type I AFPs (Figure 2A), and TXT (Figure 2C) or TCT (Figure 2D) in insect AFPs. The feature attributes, selected by our SVMGA approach also included these defining residue repeats. Some of the key SVMGA residues in representative structures of AFPs form relatively flat planes as shown by the red and yellow clustered regions in Figure 2 and 3. Additionally, our SVMGA approach identified some residues that lie at the interface between two polypeptide chains of the crystallized form used for structure determination, e.g., T13 and T24 in chain A of winter flounder antifreeze protein (PDB ID 1wfa) [35] although the active protein is a monomer (Figure 2A). Other key residues were identified by SVMGA, e.g., A8, L12, N16, and T24—all of which lie on the same side of the flat ice–binding interface which consist with the T/N/L ice–binding motif in previous work[35]. Another similar example is the β-sheet plane of chain A in 1ezg (Figure 2D). Although the TCX tri-peptide parallel strands [36] align perfectly in the dimer form, this flatter, ice–binding surface is found in the monomer and is denoted as seen by red and yellow coloration at the functional interface.

We also inspected the key residues identified in the eelpout (*Macrozoarces americanus*) type III AFP (PDB ID 1msi)—a protein that has been used in many mutagenesis studies. This protein had no homolog in any of the AFPs in the trained subsets 1, 3, 4, 5, 6, and 7 (Table 1), so the key residues were inferred using dissimilar trained sequences using the SVMGA approach. Compared with previous studies [12], [14], the SVMGA approach identified half of the known ice–binding residues at the reported interface (Figure 3). Most notable are the three residues N14, A16, and T18—mutation of these caused the greatest decreases in AFP activity[12], and the SVMGA method identified all of them. Our approach failed to recognized Q9, V20, M21, and Q44, although SVMGA identified the nearby residues, N8, T15, and L19. The mutants on residues Q9, V20 M21, and Q44 have also been reported as important residues in antifreeze activity but cause less decreasing. Residue T15 also resides at the ice–binding interface, and our method identified it as a key residue.

**Figure 4:**
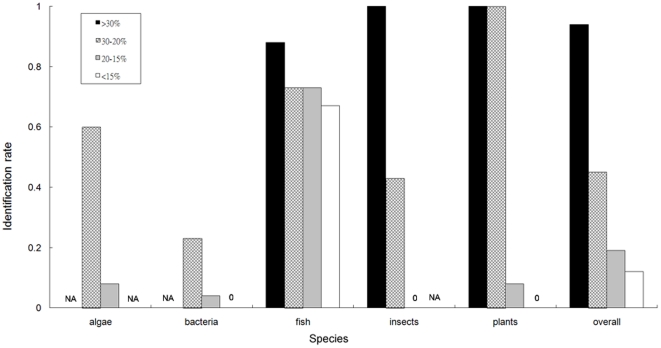
Rate of identifying the 369 AFPs from the second independent set. Each bar correlates the identification accuracy with a range of maximum SI values, found from the *y* axis of Figure 1 in specific ranges of SI for the different species.

Using the 369 AFPs in the second independent dataset (Figure 4), for which no structural information was available, the identification accuracy diminished as the evolutionary distance of a protein sequence increased from the model fish and insect sequences. For sequences with very low SI values (approximately 15∼20%), especially those from algae, bacteria, and plants, our approach gave an identification rate of approximately 20%. The identification rate of fish AFPs was around 70% accurate even when sequences with lower than 20% SI values. In fact, we believe that the features encoded in the fish and insect sequences may be used to identify AFPs from evolutionarily divergent organisms. As more sequence data for AFPs are accumulated, those data can be used to further characterize the mechanisms of cold adaptation. Finally, our approach can be used as an efficient way to obtain high throughput identification of protein function on a genome-wide scale. We have implemented our method as a web–based service, iAFP, available at http://140.134.24.89/~iafp/.
